# PARP inhibitors: clinical development, emerging differences, and the current therapeutic issues

**DOI:** 10.20517/cdr.2019.002

**Published:** 2019-09-19

**Authors:** Pooja Murthy, Franco Muggia

**Affiliations:** ^1^Department of Medicine, Maimonides Cancer Center, Brooklyn, NY 11220, USA.; ^2^New York University School of Medicine, New York, NY 10016, USA.

**Keywords:** poly-adenosyl-ribose polymerase inhibitors, poly-adenosyl-ribose polymerase inhibition, breast cancer, ovarian cancer, *BRCA*, homologous recombination deficiency, poly-adenosyl-ribose polymerase inhibitor resistance

## Abstract

Following years in development, poly-adenosyl-ribose polymerase (PARP) inhibitors continue to advance the treatment of ovarian and breast cancers, particularly in patients with pathogenic *BRCA* mutations. Differences in clinical trial design have contributed to distinct indications for each of the PARP inhibitors. Toxicity patterns are also emerging that suggest agents differ in their normal tissue tolerance - beyond what might be expected by dose variations and/or exposure to prior treatment. PARP inhibitor resistance is an increasingly relevant issue as the drugs move to the forefront of advanced ovarian/breast cancer treatment, and is an active area of ongoing research. This review examines the PARP inhibitor clinical trials that have led to approved indications in ovarian and breast cancers, PARP inhibitor targets and pharmacological differences between the PARP inhibitors, emerging mechanisms of resistance, and key clinical questions for future development.

## Introduction

The poly-adenosyl-ribose polymerase 1 (PARP1) and PARP2 enzymes are involved in base-excision repair of DNA single-strand breaks, and PARP1 also plays a role in nucleotide excision repair^[1]^ and the regulation of both nonhomologous end-joining repair^[2,3]^ and microhomology mediated end-joining repair^[4,5]^ of DNA double-strand breaks. In patients with homologous recombination deficiency (HRD), including patients with germline *BRCA1* or *BRCA2* (g*BRCA*) mutations or with non-germline HRD-positive tumors, inhibition of PARP results in production of double-strand breaks of DNA which cannot be effectively repaired. Profound susceptibility of *BRCA*-deficient or *BRCA*-mutant cells to PARP inhibition^[6,7]^ spurred the clinical development of this class of agents.

Sensitivity to platinum compounds is a feature of HRD, and a population of platinum-sensitive patients is expected to be HRD-enriched and most likely to benefit from PARP inhibition. However, platinum compounds damage DNA by several mechanisms, and cellular vulnerability to such drugs differs to variable extents from vulnerabilities to PARP inhibitors (PARPis). In addition, combinations of existing DNA-damaging drugs and PARPis, having undergone wide clinical testing, have yet to attain a therapeutic role. The current review, therefore, with some exceptions, concentrates on the use of PARPis as single agents and their emerging role in *BRCA* dysfunction related malignancies.

## Chronology of PARP inhibitor drug development in ovarian cancer

PARP’s role in DNA damage repair, and its inhibition with 3-aminobenzamide (which competes with the substrate of PARP), was a subject of study in the early 1980s^[8,9]^. However, when such strategies were explored *in vivo*, any improvement in the therapeutic index with the addition of 3-aminobenzamide to alkylating drugs was far from certain^[10]^. Nevertheless, seeking more potent PARPis than 3-aminobenzamide became the subject of structure-activity studies at Newcastle University, and subsequently in collaboration with Agouron Pharmaceuticals, AG014699 (rucaparib) was selected for pharmacologic and clinical studies by Calvert’s group^[10-12]^.

**Figure 1:**
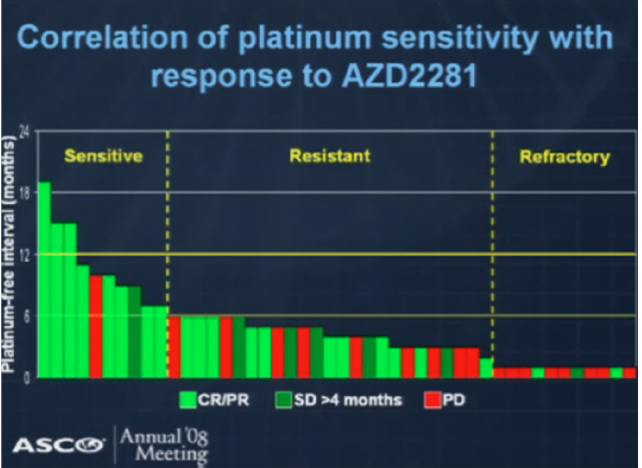
Correlation of platinum sensitivity with response to olaparib (AZD2281) in *BRCA*-mutated ovarian cancer, taken from the presentation by Fong *et al*.^[14]^ in the ASCO 2008 Annual Meeting. Longer complete and partial responses (CR/PR light green), and stable disease (SD, dark green), were seen in platinum-sensitive ovarian cancer, but also in some platinum-resistant and a brief signal in platinum- refractory ovarian cancer

In 2005, two groups reported on the remarkable cytotoxicity of PARPis towards cell lines lacking *BRCA* functionality, with Bryant *et al*.^[6]^ studying a close structural analogue to AG014699, and Farmer *et al*.^[7]^ using a Kudos compound forerunner of AZD2281, olaparib. These findings led to the concept of exploiting “synthetic lethality” - an example of how changes in two molecular pathways combine to have a lethal effect on cells although neither of them is harmful individually. In 2008, Rottenberg *et al*.^[13]^ reported on the efficacy of olaparib in *BRCA1*-deficient triple negative breast cancer mouse models, while the phase I trial of this drug was ongoing. The findings in the phase I trial clearly demonstrated single agent activity among patients with *BRCA*-mutated ovarian cancer. After expansion to include more patients with *BRCA*-mutated ovarian cancer, there was a significant association with platinum sensitivity and response to olaparib, across the platinum-sensitive, resistant and refractory subgroups, although responses were still noted in platinum-resistant patients (and even in a couple of platinum-refractory patients)^[14]^ [Figure 1]. below. This study then led to a randomized trial of pegylated liposomal doxorubicin (PLD) and olaparib in *BRCA*-mutated recurrent ovarian cancer that failed to show superiority for olaparib, perhaps because PLD over-performed in these patients who recurred within one year of first-line treatment^[15]^.

## Overview of trials leading to PARP inhibitor approval in ovarian cancer

After years in development, several PARPis have achieved indications in ovarian cancer treatment. The approved roles of PARPis in ovarian cancer fall into two main approaches: treatment of recurrent disease (the PARP inhibitor is used to shrink the tumor), and maintenance after response to platinum-based chemotherapy. The approved roles of PARPis in ovarian cancer fall into two main approaches: treatment of recurrent disease (the PARP inhibitor is used to shrink the tumor), and maintenance after response to platinum-based chemotherapy. Table 1 summarizes clinically relevant PARP inhibitor ovarian cancer trials.

**Table 1:** Selected PARP inhibitor clinical trials in ovarian cancer

Trial	Phase	Eligibility	Arms	No. of Pts	PFS (mo)	OS (mo)
Study 19 NCT00753545 (2012)^[22]^	2	Platinum-sensitive, high-grade serous ovarian cancer, received at least 2 platinum-based regimens	Maintenance olaparib 400 mg BID (capsule)	136	8.4	NSD
			Placebo	129	4.8	NSD
SOLO2/ENGOT-Ov21 NCT01874353 (2017)^[29]^	3	Platinum-sensitive, high-grade serous ovarian cancer or high-grade endometrioid cancer, received at least 2 lines of chemotherapy, with pathogenic *BRCA* mutations	Maintenance olaparib 300 mg BID	196	19.1	NM
			Placebo	99	5.5	NM
SOLO1 NCT01844986 (2018)^[27]^	3	Newly diagnosed, high-grade serous or high grade endometrioid ovarian cancer with pathogenic *BRCA* mutations	Maintenance olaparib 300 mg BID	260	Not yet reached (hazard ratio 0.30, *P* < 0.001)	NM
			Placebo	131	13.8	NM
(2014)^[18]^	2	Germline *BRCA* mutation and platinum-resistant ovarian cancer, breast cancer treated with three or more previous regimens, pancreatic cancer with previously administered gemcitabine, or prostate cancer previously treated with hormonal therapy and one systemic therapy	Treatment olaparib 400 mg BID (capsule)	298	Primary endpoint ORR: 26.2%; In pts with ovarian cancer, response rate 31.1%	Median OS in ovarian cancer pts: 16.6
ARIEL2, Part 1 NCT01891344 (2017)^[21]^	2	Recurrent, platinum-sensitive high-grade ovarian cancer, received at least 1 platinum-based regimen	Treatment rucaparib 600 mg BID	204	*BRCA* mutated: 12.8 LOH high: 5.7 LOH low: 5.2	NR
ARIEL3 NCT01968213 (2017)^[24]^	3	Platinum-sensitive, high-grade serous or endometrioid ovarian cancer, received at least 2 platinum-based regimens	Maintenance rucaparib 600 mg BID	375	10.8 (*BRCA*-mutated cohort PFS 16.6, HRD cohort PFS 13.6)	NM
			Placebo	189	5.4	NM
ENGOT-OV16/NOVA NCT01847274 (2016)^[23]^	3	Platinum-sensitive ovarian cancer, either germline *BRCA* mutation or high-grade serous histology, received at least 2 platinum-based regimens	Maintenance niraparib 300 mg daily	372	Germline *BRCA*-mutated cohort: PFS 21.0 *vs.* 5.5 Non-germline *BRCA* mutated, HRD positive cohort: PFS 12.9 *vs.* 3.8 Overall non-germline BRCA mutated cohort: PFS 9.3 *vs.* 3.9	NM
			Placebo	181		
NCT01116648 (2014)^[30]^	2	Platinum-sensitive ovarian cancer, either high-grade serous cancer or germline *BRCA* mutation	Olaparib 200 mg BID (capsule) + cediranib 30 mg daily	44	17.7	NR
			Olaparib 400 mg BID (capsule)	46	9.0	NR
NCT01081951 (2015)^[31]^	2	Platinum-sensitive, high-grade serous ovarian cancer, received up to 3 courses of platinum-based chemotherapy	Olaparib 200 mg BID (capsule) + paclitaxel 175 mg/(m^2^) + carboplatin AUC 4, then maintenance olaparib 400 mg BID (capsule)	81	12.2	NR
			Paclitaxel 175 mg/(m^2^) + carboplatin AUC 6	81	9.6	NR
TOPACIO (ovarian cancer cohort) NCT02657889 (2018)^[32]^	1/2	Recurrent, platinum-resistant/refractory ovarian cancer	Niraparib 200 mg daily + pembrolizumab 200 mg IV every 21 days	62	Primary endpoint ORR: 25%	NR

High-grade serous ovarian cancer as described here includes fallopian tube and primary peritoneal cancer. Unless otherwise specified, the olaparib formulation is the tablet formulation. The maintenance designation implies maintenance after complete or partial response to platinum-based chemotherapy. The clinicaltrials.gov identifier is included where available. Progression-free survival data is statistically significant. AUC: area under the curve; BID: twice a day; HRD: homologous recombination deficient; NM: not mature; No.: number; NR: not reported; NSD: no statistically significant difference; ORR: objective response rate; OS: overall survival; PFS: progression-free survival; Pts: patients

In the treatment of ovarian cancer, olaparib was the first PARPi to attain Food and Drug Administration (FDA) approval, with much of the initial clinical investigation efforts concentrated on women with germline *BRCA* mutations. FDA approval followed a phase 2 trial, which showed a compelling objective response rate of 34% for women with germline *BRCA* mutations and recurrent advanced ovarian cancer who progressed after 3 lines of therapy and were treated with single agent olaparib^[16]^. In this trial and other similar ones, patients with platinum-sensitive disease had a better response to olaparib than patients with platinum-resistant disease^[14,17]^. Olaparib also showed activity in platinum-resistant ovarian cancer (in patients with germline *BRCA* mutations)^[14,18]^; this distinguishes the drug from other approved PARPis that have not yet shown efficacy in platinum-resistant disease. In 2014, the FDA approved olaparib capsules for the treatment of patients with deleterious or suspected deleterious germline *BRCA*-mutated advanced ovarian cancer who were treated with three or more prior lines of chemotherapy. Later, olaparib tablets were also approved for this indication.

Rucaparib showed a similarly compelling objective response rate as single agent treatment of relapsed ovarian cancer in phase 2 trials^[19-21]^. In contrast to the olaparib trials, part 1 of the two-part phase 2 ARIEL2 trial evaluating rucaparib expanded eligibility beyond patients with germline *BRCA* mutations. Patients were classified into three subgroups; *BRCA*-mutant (either germline or somatic), *BRCA*-wild type and genomic loss of heterozygosity (LOH) high, and *BRCA*-wild type and LOH low. The primary endpoint was PFS, which was significantly longer in the *BRCA*-mutant (hazard ratio 0.27, *P* < 0.0001) and LOH high (hazard ratio 0.62, *P* = 0.011) subgroups compared to the LOH low subgroup^[21]^. Also in contrast to the olaparib data, part 1 of ARIEL2 and the other phase 2 rucaparib treatment trial, Study 10, limited enrollment to patients with platinum-sensitive recurrent ovarian cancer. Side effect profiles also differ slightly between the two drugs, affecting treatment choice (as discussed further below). In 2016, the FDA approved rucaparib for treatment of patients with deleterious *BRCA* mutation (germline and/or somatic)-associated advanced ovarian cancer who have been treated with two or more chemotherapies. The slight differences in approved treatment indications for olaparib versus rucaparib reflect differences in trial design. Ongoing trials will evaluate the efficacy of PARP inhibition in platinum-resistant ovarian cancer (including part 2 of ARIEL2), and phase 3 trials will compare PARP inhibition to standard chemotherapy, which should yield important comparison data that has been lacking from the previously mentioned nonrandomized phase II studies.

Niraparib, olaparib, and rucaparib are FDA-approved for maintenance therapy of patients with platinum-sensitive, recurrent ovarian cancer, regardless of *BRCA* mutation status, based on randomized trials that demonstrated improvement in PFS with PARPi maintenance compared to placebo following a complete or partial response to platinum-based treatment^[22-24]^. A better response was demonstrated in patients with germline *BRCA* mutations compared to the general population. Niraparib and rucaparib maintenance trials also showed that patients with deficiencies in HR, as defined by various assays, have a better response to PARPi maintenance than patients without any deficiency in DNA repair^[23,24]^. Further validation of HRD assays is ongoing. Because bevacizumab yields improved PFS in the treatment (in combination with carboplatin and either gemcitabine or paclitaxel) and maintenance of platinum-sensitive recurrent ovarian cancer when compared to standard chemotherapy alone^[25,26]^, with a trend towards improved overall survival in one trial^[26]^, it is an alternative to PARPi maintenance, especially for patients without pathogenic *BRCA* mutations or other deficiencies in HR.

More recently, PARPis are moving to the frontline maintenance setting in ovarian cancer, particularly in germline *BRCA*-mutation carriers. SOLO1 is a randomized phase 3 trial evaluating olaparib as maintenance therapy in *BRCA*-mutated patients with newly diagnosed advanced ovarian cancer who had a complete or partial response to platinum chemotherapy. Almost all patients had germline *BRCA* mutations; only 2 of 391 patients had a somatic *BRCA* mutation. The primary endpoint was PFS, which was significantly longer in the olaparib arm compared to placebo (hazard ratio 0.30, *P* < 0.001)^[27]^. While compelling and practice changing, the generalizability of this data depends in part on the definition of a deleterious germline *BRCA* mutation, especially since there are growing databases evaluating “variants of unknown significance”. As Spriggs and Longo noted in an editorial, SOLO1 did not include any information on the actual identity or distribution of the deleterious germline *BRCA* variants, which could have been useful in assigning clinical effects to specific variants^[28]^. Also, overall survival data are not yet mature, and therefore bevacizumab remains a standard maintenance option. Niraparib is also being evaluated in a frontline maintenance therapy trial, with results pending (NCT01847274). This study is not limited to patients with *BRCA* mutations; eligible patients could have either a germline *BRCA* mutation or high-grade serous histology.

## Overview of trials leading to PARP inhibitor approval in breast cancer

OlympiAD and EMBRACA were randomized phase 3 trials that compared olaparib and talazoparib, respectively, with physician’s choice chemotherapy (not including platinum chemotherapy) in patients with germline *BRCA* mutations and metastatic HER2-negative breast cancer who had received prior chemotherapy^[33,34]^. Patients must not have progressed on platinum-based chemotherapy in the metastatic setting. The primary endpoint, progression-free survival, was significantly longer in the PARP inhibitor arms, leading to FDA approval for both of these drugs. Overall survival data is still immature for EMBRACA. OlympiAD was not powered to detect a difference in overall survival, although there was a nonsignificant trend towards improvement in the olaparib arm.

## Status of trials with other PARPis

Veliparib has demonstrated less toxicity in combination with chemotherapy than the other PARPis, and continues to be evaluated in ongoing combination chemotherapy therapy trials. A phase 1 study of veliparib in combination with carboplatin and paclitaxel in advanced solid malignancies showed acceptable toxicity and promising antitumor activity^[35]^. Another phase 1 trial of veliparib this time in combination with low dose oral cyclophosphamide in refractory solid tumors and lymphomas also demonstrated acceptable toxicity and activity, but the maximum tolerated veliparib dose was much lower^[36]^. The drug has also shown single agent activity. A phase 2 study of single agent veliparib in patients with recurrent ovarian cancer who carry a germline *BRCA* mutation demonstrated an objective response rate (the primary endpoint) of 26%. 60% of the patients were platinum resistant, and these patients had a lower objective response rate compared to platinum sensitive patients (20% *vs.* 35%, respectively)^[37]^.

Talazoparib is the most potent PARP trapper of the PARPis^[38]^, and is being actively evaluated in several clinical trials. The drug is being evaluated in the neoadjuvant setting as a single agent for triple negative breast cancer, as a single agent for advanced solid tumors, in combination with temozolamide in the treatment of recurrent small cell lung cancer, alone and in combination with enzalutamide in metastatic prostate cancer, and in combination with immunotherapy (avelumab, in untreated advanced ovarian cancer) and chemotherapy [clinicaltrials.gov].

Other PARPis are in early clinical development. The PARP 1/2 and Tanykyrase 1/2 inhibitor E7449 was evaluated in a phase 1 clinical trial as monotherapy for patients with advanced solid tumors, and showed evidence of antitumor activity with low toxicity^[39]^. CEP-9722 is another PARP 1/2 inhibitor which was evaluated in a phase 1 dose-escalation trial alone and in combination with temozolomide in patients with advanced solid tumors, and showed only limited clinical activity but acceptable tolerability^[40]^. CEP-9722 was also assessed in combination with gemcitabine and cisplatin in a small dose escalation study in patients with advanced solid tumors or mantle cell lymphoma, but the study was discontinued early due to toxicities (mainly chemotherapy associated myelosuppression)^[41]^.

In 2011, there was initial excitement about the putative PARP inhibitor iniparib following encouraging results from a phase 2 trial that evaluated the drug in combination with chemotherapy in the treatment of triple negative breast cancer, but the subsequent phase 3 trial failed to demonstrate any statistically significant PFS or OS benefit^[42]^. Trials evaluating iniparib in other cancers and *in vitro* studies later indicated that iniparib does not does not function as a true PARP inhibitor^[43]^.

## Specific drug features

### PARP function, inhibitor targets, and PARP trapping

PARP enzymes catalyze poly-ADP-ribosylation (PARylation) of nuclear proteins, including themselves. Rapid PARylation at DNA damage sites is a pivotal component of the cell’s DNA damage response. Base excision repair is one of several pathways involved in the repair of single-strand DNA breaks, and relies on PARylation to recruit DNA repair complexes to the site of the break^[44,45]^.

PARP1 is also involved in maintaining genomic stability through the regulation of double-strand DNA repair processes, including the error-prone nonhomologous end-joining and microhomology-mediated end-joining processes^[2,5]^. More specifically, *in vitro* studies have found that PARP1 functions in the microhomology-mediated end-joining pathway, and that inhibition or depletion of proteins involved in this pathway, including PARP1, is synthetically lethal in cells with HRD^[46,47]^. This suggests that another mechanism for PARPi/HRD synthetic lethality is the simultaneous loss of HR and microhomology-mediated end-joining^[4]^. Overall, inhibition of PARP can induce genomic instability by shifting the balance of several DNA repair processes, which may be synthetically lethal in HRD cells.

The major substrate for the PARP enzymes is NAD+. PARPis compete with NAD+ for the PARP catalytic site. The resulting PARP inhibition affects DNA repair not just through inhibition of PARP’s catalytic activity, but also by interfering with PARP’s ability to disassociate from the damaged DNA, which is termed PARP trapping. *In vitro* studies found that PARP trapping is more cytotoxic than unrepaired single-strand breaks caused by PARP depletion^[48]^, conceivably because trapped PARP is more likely to cause stalled replication forks and double-strand DNA breaks^[49]^. Thus, PARP trapping is another explanation for the synthetic lethality of PARP inhibition in tumors with HRD.

PARP trapping potency varies considerably among the PARPis, with talazoparib demonstrating the highest PARP trapping potency^[50,51]^. Olaparib may be a weaker PARP trapper than talazoparib, and veliparib may be a weaker PARP trapper than olaparib, based mostly on *in vitro* studies^[48,50]^. However, it is important to note that efficacy and monotherapy activity of different PARPis does not correlate clearly with PARP trapping potency. Nevertheless, an individual PARPi’s trapping potency may correlate with the maximum tolerated dose and the tolerability of the drug in combination therapy (both are inversely correlated with PARP trapping potency)^[50,51]^.

**Figure 2:**
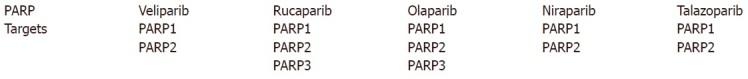
PARP inhibitor targets

The PARP family of enzymes consists of at least 17 members, of which PARP1 and PARP2 have been clearly found to participate in DNA repair. PARP1 is the best characterized and most abundant. More recently, PARP3 was found to be involved in the repair of single-strand DNA breaks^[52]^, among other functions. Detailed analysis of the differences between known PARP family members is beyond the scope of this review, and is an emerging area of research. PARP inhibitor targets include PARP1, PARP2, and PARP3; all of the clinical PARPis target PARP1 and PARP2, with some additionally targeting PARP3 [Figure 2].

It is also important to note that PARP1 and PARP2 have other functions beyond involvement in DNA break repair, which include roles in transcription, replication, modulating chromatin structure, and stabilization of replication forks. Hence, PARP inhibition has complex repercussions on cellular stability, much of which remains to be elucidated.

### Clinical findings (activity, toxicity, pharmacological features)

It is difficult to directly compare the activity of different PARPis since head-to-head studies are lacking. However, similarly designed clinical trials evaluating different PARPis have tended to show similar results. For example, the phase 3 ARIEL3 and ENGOT-OV16/NOVA trials evaluating rucaparib and niraparib, respectively, as maintenance treatment in platinum-sensitive recurrent ovarian cancer have demonstrated comparably improved PFS in the PARP inhibitor arms compared to placebo. The OlympiAD and EMBRACA trials in metastatic breast cancer, which evaluated olaparib and talazoparib, respectively, also showed a similarly improved PFS in the PARP inhibitor arms compared to physician’s choice chemotherapy.

Differences in toxicities between the PARPis, however, have emerged from these as well as other clinical trials. One cannot exclude that differences reflect not only the dosing of the agent but patient selection and prior treatment exposure to genotoxic agents. Proteome-wide profiling of the clinical PARPis also suggest that specific PARPis may have differing off target effects^[53]^, but it is not yet known whether these differences translate to unique toxicities. Common toxicities for all PARPis are fatigue, gastrointestinal toxicities (nausea/vomiting, abdominal pain, diarrhea) and cytopenias. Most of these are mild (grade 1-2). Overall, grade 3 or greater toxicities occurred in approximately 35%-56% of patients treated with the approved PARPis, of which a majority were hematological toxicities, based on data from phase 2 and 3 trials^[21-24,33,34]^. Less than 1% to 2% of patients treated with PARPis have also gone on to develop myelodysplastic syndrome or acute myeloid leukemia (AML), but it had been unclear whether this development was due to exposure to PARP inhibitor, prior chemotherapy (alkylating agents or anthracyclines), or additive effects of treatment. The recently published SOLO-1 trial evaluating frontline olaparib maintenance also showed a 1% incidence of AML in the treatment arm (compared to 0% in the placebo arm), which is worrisome because it suggests that AML may be a toxicity specific to PARP inhibitor treatment, and not related to prior chemotherapy, since these were patients treated in the frontline setting^[27]^.

Rucaparib can cause significant anemia (19%-22% of patients with grade 3 or worse anemia) and transaminitis (10%-12% of patients with grade 3 or worse transaminitis), but the transaminitis is rarely symptomatic and bilirubin does not typically increase^[21,24]^. Olaparib’s most common grade 3 toxicity is anemia, reported in 5%-22% of patients^[22,33]^, and to a lesser extent, the drug is also associated with neutropenia. Niraparib more commonly causes thrombocytopenia than the other PARPis, with grade 3 or 4 thrombocytopenia reported in 34% of patients in the ENGOT-OV16/NOVA trial, although no patients experienced grade 3 or 4 bleeding events. Grade 3 or worse anemia (25% of patients), neutropenia (20%), and hypertension (8%) were also reported^[23]^. The most frequent grade 3 or 4 toxicity for talazoparib was anemia, which was reported by 39.2% of patients enrolled in EMBRACA^[34]^. In EMBRACA, more patients in the talazoparib arm experienced grade 3-4 adverse events than patients in the standard chemotherapy arm (in contrast to olaparib in OlympiAD), although quality-of-life measurements were reassuring (talazoparib had a significant delay in the time to deterioration in health compared to the standard chemotherapy arm)^[34]^. A phase 2 trial evaluating single agent veliparib in 50 recurrent ovarian cancer patients showed a low incidence of grade 3-4 toxicities; the main grade 4 toxicity was thrombocytopenia in 2% of patients, and grade 3 adverse events were limited to fatigue in 6% of patients, nausea in 5% of patients, leukopenia in 2% of patients, and neutropenia in 2% of patients^[37]^.

Pharmacological features of the clinical PARPis are summarized in Table 2. We note that half-life informs dosing schedule, and that drug-specific metabolic pathways (involving major cytochrome P450 enzymes) tie into drug interactions.

**Table 2:** Pharmacological features of PARP inhibitors

PARP inhibitor (trade name)	Dose/formulation	Mean half-life	Metabolism	Renal dose adjustment	Hepatic dose adjustment
Veliparib (ABT-888)	Not yet approved for any indication; differing doses in clinical trials; recommended phase II dose (MTD) for single agent veliparib is 400 mg BID^[54]^	5.2 hours^[54]^	Metabolism has a secondary role in clearance (mostly renal clearance). CYP2D6 is major enzyme metabolizing veliparib, with minor contribution from CYP1A2^[55]^	Not yet available	Not yet available
Rucaparib (Rubraca)	Two 300 mg tablets BID	17-19 hours	Primarily hepatic by CYP1A2, CYP2D6, CYP3A4	CrCl ≥ 30 mL/min: no dose adjustment necessary; CrCl < 30 mL/min: has not been studied	Mild impairment: no dose adjustment necessary; Moderate-severe impairment: has not been studied
Olaparib (Lynparza)	Two 150 mg tablets BID, or three 100 mg tablets BID (replacing 400 mg capsules BID)	14.9 +/- 8.2 hours	Primarily hepatic by CYP3A4	CrCl 51-80 mL/min: no dose adjustment; CrCl 31-50 mL/min: dose reduction; CrCl < 30 mL/min: has not been studied	Mild to moderate impairment: no dose adjustment necessary; Severe impairment: has not been studied*
Niraparib (Zejula)	Three 100 mg capsules once daily	36 hours	Primarily by carboxylesterases	CrCl ≥ 30 mL/min: no dose adjustment necessary; CrCl < 30 mL/min: has not been studied	Mild impairment: no dose adjustment necessary; Moderate-severe impairment: has not been studied
Talazoparib (Talzenna)	1 mg capsule once daily	90 hours	Minimal hepatic metabolism; metabolic pathways include mono-oxidation, dehydrogenation, cysteine conjugation of mono-desfluoro-talazoparib, and glucuronide conjugation	CrCl 60-89 mL/min: no dose adjustment; CrCl 30-59 mL/min: dose adjustment; CrCl < 30 mL/min: has not been studied	Mild impairment: no dose adjustment necessary; Moderate-severe impairment: has not been studied

MTD: maximum tolerated dose; BID: twice a day; CrCl: creatinine clearance; *: For olaparib tablet formulation

## Addressing PARP inhibitor resistance

As indications for PARPis expand, and PARPis become incorporated into earlier lines of therapy, the issue of PARP inhibitor resistance becomes increasingly important and one that a clinician caring for patients with ovarian cancer will certainly have to face. There are several mechanisms of PARPi resistance, reflecting the complex interplay of PARP enzymes with DNA repair, replication, and other pathways. The field is an active area of research, and more resistance mechanisms are likely to emerge.

Mechanisms of PARP inhibitor resistance can be conceptualized as falling into one of a few categories: restoration of HR, replication fork dynamics, PARylation balance, loss of PARP1, and drug efflux. Since *BRCA* dysfunction is a key factor for the synthetic lethality of PARPis, reconstitution of *BRCA* protein and restoration of HR was early on recognized as a cause of resistance to DNA damaging agents. Incomplete data exists for PARP inhibitor resistance - but study of tumor organoids may be a way of addressing this component of resistance. Another possible feature of such reversion of *BRCA* expression is that the resistance to a PARP inhibitor may be clonal.

1. As noted above, restoration of HR: Restoration of HR abrogates the synthetically lethal effect of PARP inhibition, and can therefore confer PARPi resistance. The development of *BRCA* reversion mutations may be the most well described mechanism of HR restoration, and consequently also of PARPi resistance. Norquist *et al*.^[56]^ evaluated 46 primary and recurrent ovarian cancer specimens from patients with a history of germline *BRCA* mutations who were treated with platinum chemotherapy, and found that 28% of the recurrent ovarian cancer specimens had reversion mutations which restored the functional *BRCA* protein, compared to only 3% of the corresponding primary tumors. This percentage was significantly higher in the platinum-resistant recurrent tumors compared to the platinum-sensitive recurrent tumors (46% *vs.* 5%, *P* = 0.003). Another study evaluated patients with germline *BRCA2* mutations and advanced cancers, who had progressed on olaparib^[57]^. Pre- and post-treatment biopsies were analyzed through DNA sequencing. Secondary *BRCA2* mutations that restored the full-length BRCA2 protein were found in the recurrent tumors. Several more recent studies evaluated pre- and post-treatment tumor biopsy samples and pre- and post-treatment circulating cell-free DNA from patients with ovarian and prostate cancer, respectively, who were treated with PARPis, and found reversion mutations in *BRCA* as well as other HR genes (*RAD51C*, *RAD51D*, and *PALB2*) that correlated with progression^[58-60]^.

Besides mutations that restore *BRCA* proteins, other changes that affect the balance between HR and alternative error-prone double strand DNA break repair mechanisms could also effectively restore HR, leading to PARPi resistance. P53-binding protein 1 (53BP1) acts together with another protein, RIF1, to inhibit the end resection step of HR, antagonizing the function of *BRCA1* and promoting nonhomologous end joining (an alternative, error-prone double strand DNA repair process). Correspondingly, loss of 53BP1 has been shown to restore HR, even in cells with *BRCA* deficiency^[61,62]^. Hurley *et al*.^[63]^ evaluated archival ovarian cancer tissue specimens from a single-agent PARPi trial. The group found that PARPi responses were found exclusively in the subset of tumors with HRD, but as expected, not all the tumors with HRD responded to the PARPi. However, in the subset of tumors with HRD, the 53BP1 histochemistry score showed a strong correlation with tumor response. This study was one of the first to evaluate 53BP1 in a clinical setting, and the results highlight 53BP1’s potential role as a clinically useful biomarker to predict sensitivity to PARP inhibition.

Analogously to 53BP1, the protein encoding by the gene REV7 also antagonizes HR, and *in vitro* studies in mouse and human cell lines have shown that loss of REV7 restores HR and leads to PARPi resistance^[64]^.

Targeting upstream mediators of the DNA damage response, such as ATM and ATR, in combination with PARP inhibition, could be a strategy to circumvent the development of PARPi resistance from 53BP1 or REV7 loss.

2. Replication fork dynamics: Besides their role in DNA repair, PARP and the *BRCA* proteins are also involved in DNA replication and the stabilization of replication forks. A preclinical study showed that protection and stabilization of replication forks rescues *BRCA*-deficient stem cells, independent of any effect on HR^[65]^. This same study found that a mechanism of replication fork protection is the inhibition of nuclease recruitment to stalled replication forks, which protected the nascent DNA strands from degradation. The resulting replication fork stabilization conferred resistance to PARPis and platinum chemotherapy^[65]^. Interestingly, Hill *et al*.^[66]^ evaluated patient-derived ovarian cancer organoids, and found that a functional defect in HR in the organoids correlated with PARP inhibitor sensitivity, whereas a functional defect in replication fork protection correlated more strongly with carboplatin sensitivity. Since some patients with platinum-resistant ovarian cancer do respond to subsequent PARP inhibition (and vice versa), PARP inhibitor and platinum resistance mechanisms do not completely overlap. Differing effects of these two drug classes on DNA repair and replication fork dynamics may underlie the differences in responses.

The complex relationship between numerous factors and pathways in replication fork stabilization, including modulators of the cell cycle, is an active area of research, and therapeutic strategies addressing this mechanism of PARPi resistance are emerging (topoisomerase inhibition, cell cycle control).

3. PARylation effects: PARP’s function in DNA repair depends on its ability to catalyze PARylation of nuclear proteins. Poly-ADP-ribose glycohydrolase (PARG) is an antagonizing enzyme that digests poly-ADP-ribose moieties into ADP-ribose, and effectively “undoes” PARylation. Endogenous PARG seems to be crucial for the success of PARP inhibitor treatment, based on preclinical studies that have shown that PARG depletion partially rescues PARP1 signaling in the setting of PARP inhibitor treatment^[67]^. The same authors were also able to show that a subset of human serous ovarian and triple negative breast tumors not yet treated with PARP inhibition have PARG-negative clones, suggesting that PARG-negativity could be a biomarker predicting lack of response to PARP inhibition. Further clinical validation of this concept is needed.

4. Loss of PARP1: Immunohistochemistry studies have shown widely variable PARP1 levels in patients with ovarian and breast cancer, irrespective of *BRCA* status, but association with outcomes has been mixed^[68,69]^. Because the clinical PARPis vary in their PARP targets, chemical structures, and PARP trapping capabilities, treatment with a secondary PARPi could potentially be efficacious in a resistant tumor, but further study is needed^[70]^.

5. Drug efflux: Resistance to any drug can develop from up-regulation of drug efflux pumps (p-glycoproteins). In the case of PARPis, mouse models of *BRCA1*-deficient breast tumors treated with olaparib showed up-regulation of p-glycoproteins with ongoing treatment and maintenance. Furthermore, treatment with a p-glycoprotein inhibitor (tariquidar) following relapse on olaparib re-sensitized the tumor to olaparib and led to tumor regression^[13]^. Because p-glycoprotein inhibitors lack specificity and are associated with significant toxicity, targeting upstream regulators of these drug efflux pumps may be a better tolerated strategy, and is being evaluated^[70]^.

Many other resistance mechanisms are emerging, and with them, strategies to evade resistance, or to use gene or protein expression as predictive biomarkers, are also developing. We note that epigenetic changes, including *BRCA* gene methylation, and microRNA and long non-coding RNA regulation, have been found to correlate with PARPi resistance^[71,72]^, and could be developed into biomarkers. Since HR is highly cell cycle dependent (depends on the sister chromatid for DNA repair), regulation of the cell cycle may be a way to re-establish an HRD state in tumors with HR reversion mutations. Inhibition of WEE1, a cell cycle regulator, had activity in some patients with *BRCA*-deficient tumors in a phase 1 clinical trial^[73]^. Combination therapies of cell cycle regulators with PARP inhibition may therefore hold promise as a way to circumvent PARPi resistance, if toxicities are manageable. Targeting complimentary DNA repair pathways, such as the microhomology-mediated end-joining pathway, together with PARP inhibition could represent another strategy to prevent or mitigate resistance, by augmenting synthetic lethality in a tumor with HRD.

## Future perspectives

### Biomarkers

There is much interest in evaluating biomarkers for PARP inhibition, both because intrinsic or developing resistance are concerns, and also to more precisely expand the eligible patient population beyond patients with *BRCA* mutations. So far, the main clinically validated biomarker for response to PARPis is the presence of germline or somatic *BRCA* mutations. Assays for HRD, as used in the ARIEL and NOVA trials, require further clinical validation before they are used in clinic, and continue to be evaluated, such as in the QUADRA trial^[74]^. Many other potential biomarkers are emerging, as above, and may predict for PARP inhibitor resistance. Correlative studies in PARP inhibitor trials should yield valuable data on these emerging biomarkers. An ongoing clinical trial is evaluating long-term responders on olaparib (NCT02489058), and may show important information on predictive factors for response.

A central difficulty in the clinical use of biomarkers for PARP inhibitor response is the evolving nature of the tumor; a marker may represent genomic scarring, or evidence of prior repair deficiency, and may not represent the current state and capabilities of the tumor. Therefore, frequent genomic assessment of the tumor may be required to dynamically assess resistance and fully inform treatment decisions. Because tissue biopsies require invasive procedures, “liquid biopsies”, or plasma circulating tumor DNA, would capture emerging biomarkers and may provide sufficient information to guide treatment decisions in the future^[59,60]^.

### Monotherapy versus combination therapy

Preclinical and some clinical data indicate that immune checkpoint inhibition may synergize with PARP inhibition in tumors with HRD, and that tumors with defective DNA repair are especially sensitive to immunotherapy^[75-77]^. One study evaluated *BRCA1*-deficient mice with triple negative breast cancer and found that cisplatin combined with dual checkpoint blockade augmented antitumor immunity, attenuated tumor growth, and improved survival. *BRCA1*-deficient tumor models were also found to have an increased somatic mutation burden, greater number of tumor-infiltrating lymphocytes, and increased expression of immunomodulatory genes (*PD-1* and *CTLA4*) compared to *BRCA*-wild type tumor models^[78]^. Compelled by these and other data, several ongoing clinical trials are evaluating PARPis in combination with immune checkpoint inhibitors in breast and ovarian cancers. Angiogenesis inhibitors (cediranib) in combination with PARP inhibition have demonstrated encouraging activity in a phase 2 platinum-sensitive ovarian cancer clinical trial^[30]^, with notable activity even in patients without *BRCA* mutations. Several other ongoing clinical trials are evaluating angiogenesis inhibitors in combination with PARP inhibition in ovarian and other cancers.

Novel agents, such as ATM, ATR, and WEE1 inhibitors, are also being evaluated in combination with PARPis, as part of strategies to evade PARP inhibitor resistance and augment synthetic lethality, as described in the above PARP inhibitor resistance sections. Combinations of PARPis plus chemotherapy, and PARPis plus signal transduction inhibitors such as PI3 kinase inhibitors (NCT01623349) are additionally being studied.

Based on earlier data that has motivated many of these combination therapy trials, we may anticipate positive signals from at least a few of the ongoing studies. Ultimately, however, the tolerability of combination regimens will need to be assessed and may be an impediment to eventual use in the clinic.

### Sequencing PARP inhibitor treatment

An unresolved question in the treatment of *BRCA*-associated advanced breast cancer is how to sequence PARPis with platinum chemotherapy, since both these agents are active in the disease and work through DNA damage. Ovarian cancer data demonstrates olaparib responses even in platinum-resistant patients, and the phase 2 ABRAZO trial that evaluated talazoparib in patients with advanced breast cancer and germline *BRCA* mutations showed a PFS of 4 months for patients who had progressed at least 8 weeks after the last dose of platinum chemotherapy^[79]^. Therefore, treating with a PARP inhibitor following progression on platinum-based chemotherapy has some basis. There is less data on treating first with a PARP inhibitor followed by platinum chemotherapy. A phase 2 trial of patients with germline *BRCA* 1/2-mutated metastatic breast cancer assessed single-agent veliparib, another PARPi, followed by veliparib plus carboplatin at disease progression. The post-progression treatment with veliparib and carboplatin at the maximum tolerated doses (150 mg BID, and AUC of 5, respectively) yielded minimal benefit; only one patient out of 30 had a response^[80]^. However, since PARPis are reasonably well tolerated, treatment with a PARP inhibitor early in the disease course may be the preferred approach for some patients. Overall, the optimal sequence of DNA damaging agent treatment in breast cancer still needs to be determined.

The activity of PARPis and platinum chemotherapy following progression on the other agent also needs to be further investigated in ovarian and other cancers. Differences in resistance mechanisms between platinum compounds and PARPis could inform these treatment decisions in the future, but at this point, requires further study. Even less is known about the potential activity of a specific PARP inhibitor following progression on another PARP inhibitor, but because the clinical PARPis have different chemical structures, targets, trapping potency, and other off-target effects, this would be a valuable clinical question to explore.

## Declarations

### Copyright

© The Author(s) 2019.
